# Role of Multicellular Aggregates in Biofilm Formation

**DOI:** 10.1128/mBio.00237-16

**Published:** 2016-03-22

**Authors:** Kasper N. Kragh, Jaime B. Hutchison, Gavin Melaugh, Chris Rodesney, Aled E. L. Roberts, Yasuhiko Irie, Peter Ø. Jensen, Stephen P. Diggle, Rosalind J. Allen, Vernita Gordon, Thomas Bjarnsholt

**Affiliations:** a Department of Immunology and Microbiology, University of Copenhagen, Copenhagen, Denmark; b Department of Clinical Microbiology, Rigshospitalet, Copenhagen, Denmark; c Center for Nonlinear Dynamics and Department of Physics, The University of Texas at Austin, Austin, Texas, USA; d School of Physics and Astronomy, University of Edinburgh, Edinburgh, United Kingdom; e Centre for Biomolecular Sciences, School of Life Sciences, University of Nottingham, Nottingham, United Kingdom; f Department of Biology and Biochemistry, University of Bath, Bath, United Kingdom; g Institute for Cellular and Molecular Biology, The University of Texas at Austin, Austin, Texas, USA

## Abstract

In traditional models of *in vitro* biofilm development, individual bacterial cells seed a surface, multiply, and mature into multicellular, three-dimensional structures. Much research has been devoted to elucidating the mechanisms governing the initial attachment of single cells to surfaces. However, in natural environments and during infection, bacterial cells tend to clump as multicellular aggregates, and biofilms can also slough off aggregates as a part of the dispersal process. This makes it likely that biofilms are often seeded by aggregates and single cells, yet how these aggregates impact biofilm initiation and development is not known. Here we use a combination of experimental and computational approaches to determine the relative fitness of single cells and preformed aggregates during early development of *Pseudomonas aeruginosa* biofilms. We find that the relative fitness of aggregates depends markedly on the density of surrounding single cells, i.e., the level of competition for growth resources. When competition between aggregates and single cells is low, an aggregate has a growth disadvantage because the aggregate interior has poor access to growth resources. However, if competition is high, aggregates exhibit higher fitness, because extending vertically above the surface gives cells at the top of aggregates better access to growth resources. Other advantages of seeding by aggregates, such as earlier switching to a biofilm-like phenotype and enhanced resilience toward antibiotics and immune response, may add to this ecological benefit. Our findings suggest that current models of biofilm formation should be reconsidered to incorporate the role of aggregates in biofilm initiation.

## IMPORTANCE

During the past decades, there has been a consensus around the model of development of a biofilm, involving attachment of single planktonic bacterial cells to a surface and the subsequent development of a mature biofilm. This study presents results that call for a modification of this rigorous model. We show how free floating biofilm aggregates can have a profound local effect on biofilm development when attaching to a surface. Our findings show that an aggregate landing on a surface will eventually outcompete the biofilm population arising from single cells attached around the aggregate and dominate the local biofilm development. These results point to a regime where preformed biofilm aggregates may have a fitness advantage over planktonic cells when it comes to accessing nutrients. Our findings add to the increasingly prominent comprehension that biofilm lifestyle is the default for bacteria and that planktonic single cells may be only a transition state at the most.

## INTRODUCTION

Biofilms are three-dimensional (3D) communities of interacting unicellular organisms (1). In a biofilm of supposedly genetically identical clones, the constituent cells develop differentiated patterns of gene expression and growth (2, 3). Differentiation is often linked to the positioning of cells in the biofilm structure, and the spatial location of cells also affects resource availability and intercellular contacts (4).

The initiation of *in vitro* biofilm formation has traditionally been thought to be due to random attachment of single cells to a surface; these cells then divide and develop into mature, three-dimensional biofilms (5, 6). However, when cells disperse to seed new biofilms, detachment can occur by dispersal of single motile cells or by the sloughing off of large aggregates of cells (7–9). Both single cells and multicellular aggregates can then go on to initiate new biofilms. For aquatic biofilms, the enhanced stickiness and surface conditioning of planktonic multicellular aggregates have been shown to increase the attachment of bacteria to a surface in early biofilm initiation (10). Greater stickiness and surface conditioning may be considered a quasiphenotypic physiological property resulting from greater levels of organic polymers and colloids. Similarly, an increased tendency toward aggregation, likely a proxy for greater stickiness, has also been associated with increased biofilm formation for *Pseudomonas aeruginosa* (11, 12).

Three-dimensional bacterial aggregates found in liquid batch cultures of *P. aeruginosa* can have diameters of 10 to 400 µm and can constitute up to 90% of the total biomass of the culture (13). In contrast, individual *P. aeruginosa* cells are rod shaped and have a size of ~1 µm by 2 µm. Thus, multicellular *P. aeruginosa* aggregates, when they attach to a surface, can extend significantly into the vertical dimension, away from the attachment surface. Moreover, aggregates are structurally and physiologically distinct from single cells. Yet, how these structural contrasts impact the seeding and growth of new biofilms is not known. This constitutes a significant gap in our understanding, since a biofilm seeded from an aggregate may develop very differently from a biofilm seeded by single cells.

To investigate the influence of preformed aggregates on biofilm development, we first preformed computer simulations of biofilm development from aggregates and single cells using an individual-based model and subsequently measured the relative growth of single cells and aggregates of *P. aeruginosa* during *in vitro* biofilm development in flow cells. Using biomass accumulation as a measure of growth fitness, we found that the relative fitness of aggregates was highly dependent upon the surrounding number and density of single cells on the surface, which we use as a proxy for the level of competition for growth resources. We found that when the initial surrounding density of single cells is low, aggregates are less fit than single cells, yet when the surrounding density of single cells is high, aggregates are more fit than single cells are. Our results show that in highly competitive environments, the 3D configuration in which cells land on a surface can greatly affect their relative fitness, both in the earliest stages of biofilm development and during long-time development. Our work calls for a modification of the traditional model of biofilm development to take into account the impact of preexisting cell aggregates. This opens new avenues to understanding the evolution and ecology of biofilms in the environment and in chronic infections such as cystic fibrosis, chronic wounds, and implant-related infections.

## RESULTS

### Simulated fitness of aggregates versus single cells in the initial formation of biofilms.

To investigate the fate of initial aggregates versus single cells during biofilm development, we first carried out individual-based computer simulations, in which biofilms were grown from aggregates surrounded by competing single cells. By varying the density of single cells surrounding the aggregate, we were able to vary the extent of competition in our simulations.

**FIG 1:**
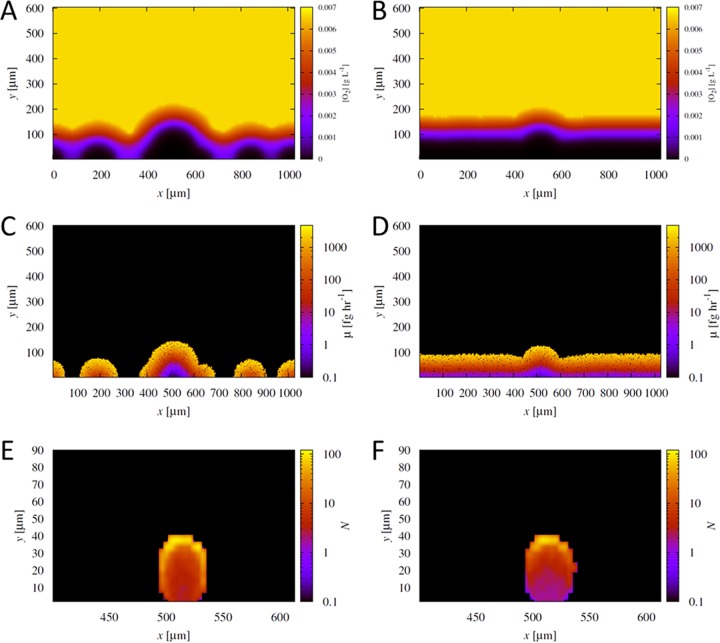
Biofilm morphology shapes the oxygen concentration profile, with the fittest cells initially located at the top. (A and B) The oxygen concentration (in grams liter^−1^) (right-hand *y* axes) in a sample simulation after 30 h of growth of cells at low density (0.01 cell µm^−1^) (A) and high density (0.5 cell µm^−1^) (B). *x* and *y* (both in micrometers) are the spatial dimensions of the simulation domain. (C and D) Growth rate (μ) (right-hand *y* axes) for the resulting populations after 30 h of growth at low density (0.01 cell µm^−1^) (C) and high density (0.5 cell µm^−1^) (D). (E and F) 2D histograms representing the number of progeny, *N* (right-hand *y* axes), produced after 30 h of growth by individual bacteria as a function of their initial location in the aggregate: low density (0.01 cell µm^−1^) (E) and high density (0.5 cell µm^−1^) (F). These distributions were averaged over 40 simulations for each aggregate. Note that the gradient in the number of progeny is so large that a log scale is used for visualization purposes.

In our simulations, we use oxygen as the growth-limiting resource (see “The growth-limiting resource may be oxygen” below). Figure 1A and B show the oxygen concentration profile after 30 h of growth in the low-density (Fig. 1A) and high-density (Fig. 1B) regimes. Comparing Fig. 1A and B with Fig. 1C and D, which show the growth rates of individual cells as a function of their position in the growing biofilm, it is clear that the oxygen concentration profiles are influenced by the morphology of the growing biofilms. In the growing biofilms, we see that oxygen is depleted in the deeper regions; this oxygen-deprived layer emerges because faster growing cells at the top (Fig. 1C and D) consume oxygen faster than it can diffuse to the deeper regions. This in turn leads to further heterogeneity in individual cell growth rates (Fig. 1C and D), resulting in two distinct layers of growth activity: an outer layer of metabolically active cells and an interior region of inactive cells. Simulation snapshots of biofilms formed after 10, 30, and 120 h of simulated growth are shown in Fig. S1 in the supplemental material.

**FIG 2:**
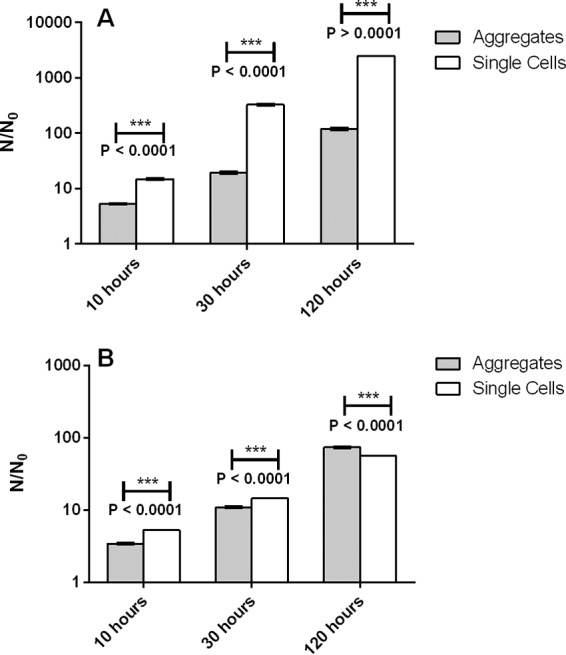
Simulations reveal that aggregates are relatively fitter than single cells at high density of competing cells on the surface and over long times. (A and B) Accumulated biomass normalized to initial biomass (*N*/*N*_0_) after 10, 30, and 120 h for single cells and for aggregates initiated at a low starting density (0.01 cell µm^−1^) (A) and a high starting density (0.5 cell µm^−1^) (B). For biofilms that were initiated at a low density, single cells produce more progeny than do cells in aggregates at all measured times. For biofilms that were initiated at a high density, single cells are more fit for early growth but aggregated cells produce more progeny than single cells do after 120 h.

We compared the fate of cells that originated in aggregates with that of initially unaggregated cells after 120 h of simulated growth (see Materials and Methods). To this end, we used the number of progeny per initiating cell, *N*/*N*_0_ (where *N* is the number of cells at time *t*, and *N*_0_ is the initial number of cells) as a measure of fitness. Our simulation results show enhanced performance of the aggregates compared to unaggregated cells with increasing initial density. When the initiating density of surrounding single cells is low, initially unaggregated cells show higher fitness than those in an aggregate (Fig. 2A). However, when the initiating density of surrounding cells is high, cells in an aggregate perform better than the initially unaggregated cells over long times (Fig. 2B). This change in the relative fitness reflects a decrease in the fitness of the single cells as their density increases, rather than any substantial change in fitness of the aggregated cells (see Fig. S2 in the supplemental material).

### The interplay between competition and spatial structure determines the relative fitness of aggregates.

Our simulations show that the aggregate produces more progeny per initial cell than do its initially unaggregated counterparts only when competition for resources is high (and over long times, when space and resources become limited due to larger numbers of cells). Why does increased competition favor the aggregate? Closer inspection of Fig. 2A and B reveal that, in fact, the fate of the aggregate is little affected by the increase in cell density on the surface. For instance, *N*/*N*_0_ for the aggregate at 30 h decreases from ~14 (14.27) to ~11 (10.94) when going from low density to high density. However, the decrease in *N*/*N*_0_ for the initially unaggregated cells over the same time period is much larger (~328 to ~15). To analyze the fate of the aggregate in more detail, Fig. 1E and F show the average number of progeny produced by cells in the initial aggregate as a function of their initial position. As shown previously (14), the initial position of a cell within an aggregate has a strong effect on the number of progeny it produces; cells in the interior produce fewer progeny than those initially located at the upper edges (Fig. 1E). With increasing density of surrounding cells on the surface (Fig. 1F), we see that cell fate is even more heterogeneous, with the most prolific cells now localized in the highest portion of the aggregate. Thus, although the increased density of unaggregated cells on the surface increases the level of competition for space and resources, our simulations reveal that the few cells initially located at the top of the aggregate dominate the fate of the aggregate at all levels of competition.

### The relative fitness of aggregates and single cells depends on the initial cell density.

**FIG 3:**
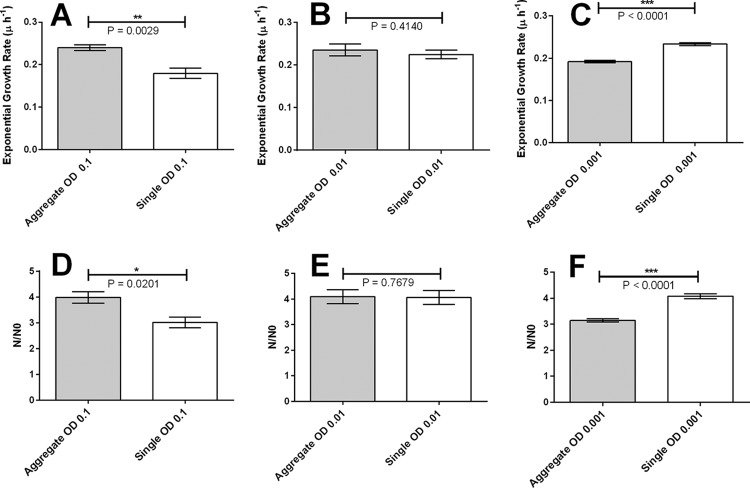
In *in vitro* growth in flow cells, at low inoculum density, aggregates are less fit than single cells; at high inoculum density, aggregates are more fit than single cells. (A to C) Fitted exponential growth rates during the first 9 h of growth for initially aggregated cell and initially single-cell populations, starting with different cell densities in the inocula. Inoculum optical densities (OD) of 0.1 (A), 0.01 (B), and 0.001 (C) were used. (D to F) Measured biomass ratio *N*/*N*_0_ after 9 h of growth. Inoculum ODs of 0.1 (D), 0.01 (E), and 0.001 (F) were used. Values are means ± standard errors of the means (SEM) (error bars).

To test our *in silico* simulation predictions, we investigated experimentally the degree to which seeding with single cells versus preformed aggregates gave rise to different patterns of biofilm growth. We inoculated flow cells with an overnight culture containing both planktonic cells and aggregates. By varying the cell density of the inoculum from an optical density (OD) of 0.001 to 0.1, we were able to vary the seeding density of single cells and thus the level of competition for growth resources on the coverslip surface of the flow cell. We imaged single cells and aggregates over the first 9 h of growth, and from these data, we obtained growth rates based on the change in biomass over time. Due to small variations in growth rates from experiment to experiment, we concentrated solely on the relative fitness of single cells and aggregates within the same experiment and did not compare absolute growth rates between experiments. As predicted by our simulations, we found that the relative fitness of aggregates compared to that of single cells depends markedly on the density of seeding cells. At a low inoculum density (OD of 0.001), aggregates grew (0.1920 ± 0.0126 division h^−1^) at a significantly lower rate than single cells grew (0.23 ± 0.0159 division h^−1^) (*P* < 0.0001). At a medium inoculum density (OD of 0.01), there was no difference in growth rate between aggregates (0.2349 ± 0.028 division h^−1^) and single cells (0.224 ± 0.031 division h^−1^) (*P* = 0.414). At a high inoculum density (OD of 0.1), cells in aggregates grew faster (0.24 ± 0.017 division h^−1^) than single cells (0.1795 ± 0.04 division h^−1^) (*P* = 0.0029). Growth rates for cells in aggregates and single cells are plotted in Fig. 3A to C, and the results of exponential fits and significance tests are summarized in Table S1 in the supplemental material.

Although growth of single cells and aggregates over the first 9 h of growth appears exponential, fitting with a single exponent makes the implicit assumption that all cells within an aggregate are growing at the same rate. To avoid this assumption, we also describe growth in terms of the number of progeny per initial cell, *N*/*N*_0_. Here, *N* is the biomass after 9 h of growth, and *N*_0_ is the initial biomass. We find that *N*/*N*_0_ is greater for the aggregates in the high-density treatment (Fig. 3D) and greater for single cells at low density (Fig. 3F). Thus, the *N*/*N*_0_ representation captures the same density-dependent advantage for aggregates as the exponential growth rate representation. Therefore, our results show that there is a relative disadvantage to growing in an aggregate at low competition and a relative advantage to growing in an aggregate at high competition.

**FIG 4:**
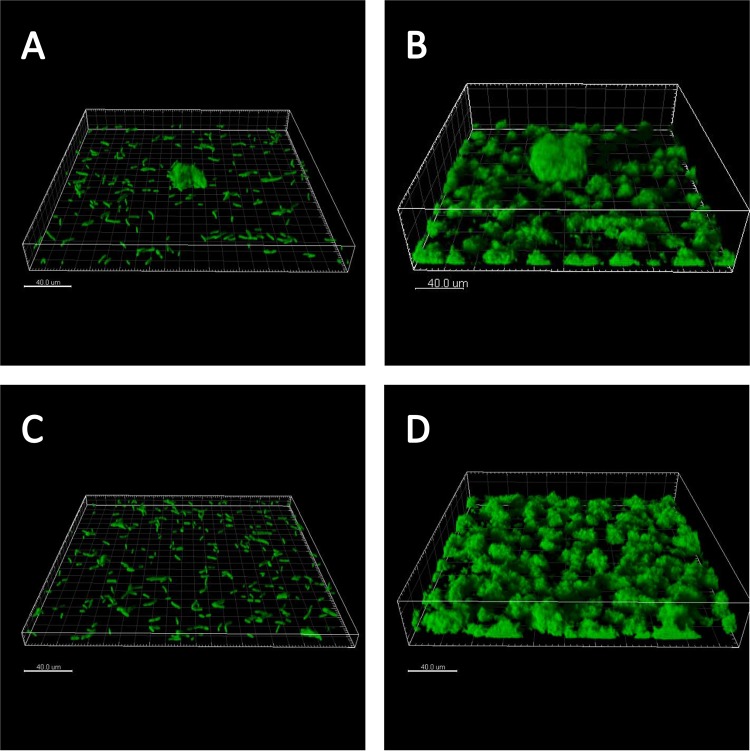
The presence of multicellular aggregates at the start of biofilm growth is reflected in the structure of the biofilm a day later. Shown are perspective projections created from confocal microscope z-stacks of *Pseudomonas aeruginosa* biofilms. (A) Single cells attached to the surface at 0 h. (B) A preformed aggregate surrounded by single cells on the surface at 0 h. (C) Biofilm descending from single cells from panel A. (D) After 24 h of growth, a large biofilm structure descending from the preformed aggregate shown in panel B surrounded by biofilm descending from single cells.

To explore the dynamics of competition and the spatial distribution of cells within the biofilm over longer time periods, we followed the growth of aggregates and single cells in the flow cell up to 99 h but focused primarily on the first 24 h after inoculation. We found that areas that were seeded with an aggregate developed a corresponding large vertical protrusion above the surrounding biofilm lawn (Fig. 4D). Areas that were initially seeded by only single cells developed into a much more homogeneous, unstructured lawn (Fig. 4C).

### The fitness of cells is enhanced by higher spatial positioning.

**FIG 5:**
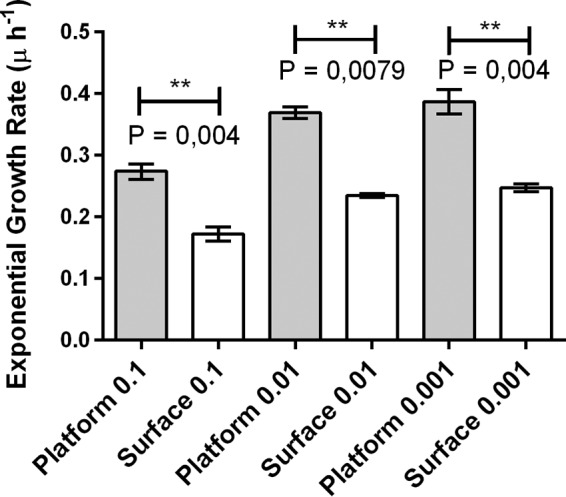
Fitted exponential growth rates for the first 9 h of growth of single cells of *P*. *aeruginosa* PAO1 either on the surface or elevated 100 µm above on a glass platform. The fractional relative fitness (*w*) is about 0.5 for all densities evaluated, indicating that cells on the step consistently have a growth advantage over cells on the surface. Low density was an initial inoculum OD of 0.001. Medium density was an initial inoculum OD of 0.01. High density was an initial inoculum OD of 0.1. Values are means ± SEM (error bars).

Taken together, our simulation results and experimental results suggest that height above the surface of the flow chamber may be a crucial factor in our experimental setup; for example, in our simulations, cells that are at the top of an aggregate produce more progeny. This suggests that, in general, cells positioned above the surface of the flow chamber should outperform cells that are positioned closer to the surface. To test this, we measured the growth of single cells positioned on a glass step 100 µm above the chamber surface and compared it to the growth of single cells on the chamber surface. Indeed, we found that, regardless of the initial density, cells positioned on the step (see Materials and Methods; see Fig. S5 in the supplemental material) grew faster than single cells on the chamber surface (*P* = 0.004, *P* = 0.0079, and *P* = 0.004 for ODs of 0.1, 0.01, and 0.001, respectively) (Fig. 5; see Table S2 in the supplemental material). Furthermore, we also performed a series of simulations in which we eliminated the height advantage of the aggregate by surrounding it by a pregrown layer of competitor cells of equal height (see Materials and Methods and supplemental material). As expected, the cells in the aggregate no longer outperformed the single cells in these simulations.

### Better substrate access at the top of aggregates can lead to growth instability.

**FIG 6:**
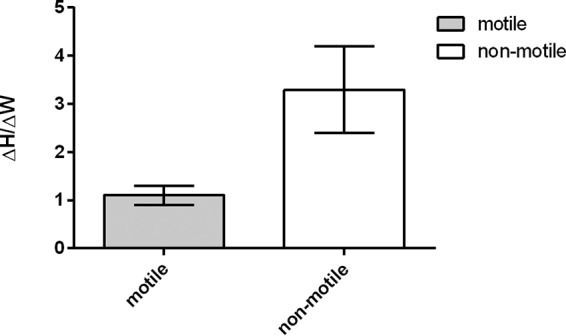
Change in aspect ratio for aggregates after 6 h of growth at high competition (OD of 0.1). Mean change in aspect ratio (Δ*H*/Δ*W* where *H* represents height and *W* represents width) for motile (WT PAO1) and nonmotile (PAO1 Δ*pilA*) aggregates. Values are means ± SEM (error bars).

Our simulations show that an aggregate contains a subpopulation of slow-growing cells in its center and a subpopulation of fast-growing cells at the top of the aggregate; this differentiation arises due to spatial gradients in the growth resource. Since cells at the top of an aggregate grow faster, this might suggest that, over time, the shape of aggregates should become less spherical and more prolate spheroid. We checked for this change in aspect ratio in our experiments by measuring the height and width (at half height) of aggregates at the beginning of an experiment and 6 h later. We then calculated the fold change in aspect ratio over the 6-h time period, Δ*H*/Δ*W*, where Δ*H* is the fractional change in height and Δ*W* is the fractional change in width. A value of 1 for Δ*H*/Δ*W* would signify symmetrical growth in all directions, while a value greater than 1 would signify aggregates becoming more prolate spheroid. To measure the fold change in aspect ratio, we used wild-type (WT) *P*. *aeruginosa* PAO1 aggregates and twitching motility knockout Δ*pilA* PAO1 aggregates at a high cell density (Fig. 6). The change in aspect ratio is greater than 1 for the Δ*pil* mutant, but not for the twitching-motile WT. This suggests that, as our model predicts, cells at the top of the aggregate are growing more quickly than cells in other parts of the aggregate—but also that twitching-capable cells rearrange themselves to reduce the local cell density in the aggregate.

### The growth-limiting resource may be oxygen.

**FIG 7:**
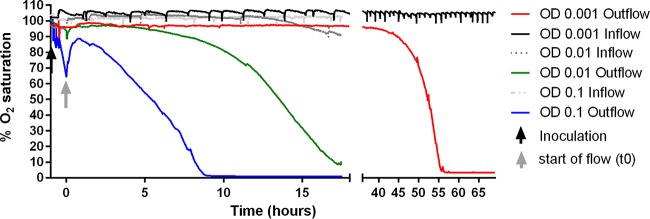
Oxygen content in inflow and outflow of media to the flow cells inoculated with bacterial cells at an OD of 0.001, 0.01, or 0.1. After inoculation, the cells were left without flow for 1 h before starting the flow at *t*_0_. From *t*_0_ to *t*_9_, the AUC values for outflow medium were 546.4, 854.7, and 874.3 for 0.1, 0.01, and 0.001 (% O_2_ saturation * h), respectively.

We found no effect on our results upon varying the concentration of the carbon source over 4 orders of magnitude, indicating that carbon is likely not the limiting growth resource. In contrast, by measuring oxygen concentrations in the inflow and outflow of our flow cells, we find that oxygen, the only electron acceptor present in our media, becomes limited within the first 9 h of growth for our high-density inoculation. The values for the area under the curve (AUC) for oxygen in the outflow medium were 546.4, 854.7, and 874.3 (% O_2_ saturation * h) for flow cells inoculated with cells at ODs of 0.1, 0.01, and 0.001, respectively, for the first 9 h of growth. Oxygen is thus very limiting for growth in our flow cells, which were inoculated with cells at an OD of 0.1. For flow cells inoculated with cells at an OD of 0.01, oxygen is somewhat limiting. For the flow cells inoculated with cells at an OD of 0.001, oxygen levels in the outflow are not limiting within the 9-h time frame in which growth was measured. This strongly points toward oxygen as a growth-limiting resource in our experiments at a high cell density (OD of 0.1) and to a lesser extent at a medium cell density (OD of 0.01). As the outflow medium content is close to 100% O_2_ saturation for the flow chambers inoculated with cells at a low density over all 9 h of measurement, we anticipate no significant competition for oxygen (Fig. 7).

## DISCUSSION

Nonattached biofilm aggregates arise in the liquid phase of *in vitro* bacterial cultures and in natural liquid environments, and these aggregates are likely to attach often to surfaces. Despite this, little is known about the role aggregates play in biofilm development compared to single cells. This study examined the biofilm growth dynamics that arise when a biofilm is seeded from a preformed aggregate using both *in silico* simulation and a widely used *in vitro* biofilm reactor system. Our simulations deliberately neglected many biological mechanisms, including exopolysaccharide production, cell-cell signaling, and cell detachment. Thus, any phenomena that arise in our simulations can be attributed solely to cell growth, competition for growth resources diffusing from above, and mechanical interactions between cells. Therefore, we attribute the change in relative fitness of the aggregates with the level of competition to the interplay between the spatially structured environment and the spatial distribution of cells in the aggregate.

We found that aggregates have a fitness advantage over single cells when competition for resources is high since the elevated position of cells at the top of the aggregate gives these cells better access to growth resources. However, when competition is low, the single cells have access to resources that is comparable to that of cells at the top of the aggregate and better than that of cells in the aggregate interior. As a result, when competition is low, single cells are more fit than aggregates are. This shows that the relative fitness of aggregates depends markedly on the density of surrounding single cells, i.e., on the level of competition for growth resources. When competition between aggregates and single cells is low, an aggregate has a net growth disadvantage because the aggregate interior has poor access to growth resources. However, if competition is high, aggregates have a higher net fitness, because extending vertically above the surface gives cells at the top of the aggregates better access to growth resources. Our findings suggest that we should reconsider our models of biofilm formation to incorporate the role of aggregates, because current models focus only on growth that is initiated from individually attached cells.

Upon comparing results for short, intermediate, and long periods of biofilm growth, we found that the fitness of aggregates, relative to that of single cells, increases with time (Fig. 2; see Fig. S4 in the supplemental material). Thus, the outcome of competition between initially aggregated and single cells is dependent on time. As biofilms develop, the descendants of aggregates tend to dominate (Fig. 2 and 4), since competition for growth resources becomes more intense as the total biofilm biomass increases. This suggests that, in the long term, if competition for growth resource is the sole pressure on cells, structures such as aggregates that protrude into the third dimension, and thus have better access to growth resources, will always be favored over single cells and their descendants.

We know from previous investigations, and have confirmed here, that overnight liquid batch cultures of *P. aeruginosa* contain both single cells and multicellular aggregates (13, 15). These multicellular aggregates range in size from 10 µm to several hundred microns in diameter (13). After inoculating a flow cell with an overnight culture of *P. aeruginosa* PAO1, one typically finds some fields of view that are seeded only with single cells (Fig. 4A) and other fields of view that are seeded with an aggregate surrounded by single cells (Fig. 4B). We expect to see cells on the surface of an aggregate grow faster than those in the interior because the latter have restricted access to growth resources (4, 16).

The results of our *in vitro* flow cell experiments confirmed the findings from our simulations, showing competition-dependent fitness advantages for aggregates over single cells. The density of surrounding single cells determines the relative impact of the spatial distribution of cells in the aggregate. Under conditions of low competition, single cells and cells on the surface of an aggregate have free access to resources and can grow unrestricted, whereas cells in the aggregate interior have less access to resources and grow more slowly. This puts the cells in the aggregate at an overall fitness disadvantage compared to single cells. However, as the level of competition among cells on the chamber coverslip surface increases, single cells on average produce fewer progeny. Cells at the top of aggregates are elevated above the level of the surrounding single cells and are closer to untapped growth resources. In this scenario, cells within aggregates have a higher relative fitness than single cells.

In addition, our *in vitro* step experiment shows an enhanced growth rate for cells elevated above the surface of the flow chamber compared to single cells positioned on the surface of the flow chamber. This supports our hypothesis that cells on the top of an aggregate have a growth advantage over single cells, due to their height. This growth advantage of the cells at the top of the aggregate compensates, under conditions of high competition, for the slower-growing cells contained within the aggregates.

**FIG 8:**
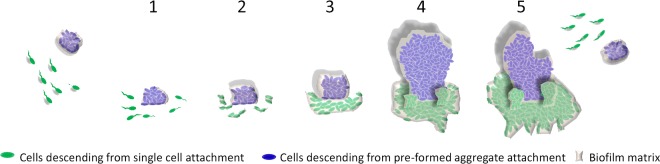
Proposed revision of biofilm development. The five classical stages of development in the presence of a preformed, multicellular aggregate are shown. In stage 1, the surface can be seeded either by single cells in a planktonic phenotype or by a preformed aggregate. In stage 2, the single cells attach irreversibly, and the aggregated population grows. In stage 3, the biofilm matures with complete matrix. Descendants from the aggregate population reach out in an elevated structure. Stage 4 is the mature structured biofilm. The descendants of the aggregate tower several times higher than any surrounding structures descended from single cells. In stage 5, dispersal of single cells and sloughing off of biofilm aggregates occur.

Our results suggest a new model for early biofilm development, in which seeding of the biofilm from preformed aggregates plays a major role (Fig. 8). Our results also imply that, when competition for resources is the main selective force, structures that initially protrude above a surrounding lawn of cells will be favored. These results raise a number of evolutionary questions. Why might evolution favor the formation of multicellular aggregates, given that our simulations show that only cells at the top of aggregates produce large numbers of progeny and many of the constituent aggregate cells sacrifice their own fitness for the benefit of these cells? Aggregation may be maintained by kin selection, a process by which traits are favored because of their beneficial effects on the fitness of close relatives, such as those cells at the top of aggregates (17, 18). Previous simulation work has also shown height-related fitness advantages and kin selection during biofilm development, suggesting that strains of bacteria that produce aggregation-promoting extracellular polymeric substances (EPS) gain a fitness advantage in biofilms by pushing their progeny upwards into the medium (19). Our work supports this view, while also showing that such fitness advantages can arise by aggregation as well as by traits such as EPS production.

While our work suggests one possible advantage of aggregate formation, we note that cells in an aggregate may also incur other benefits, especially in an *in vivo* infection. Aggregates exhibit many of the same phenotypes as surface-attached biofilms (15), meaning that they demonstrate increased antibiotic tolerance, resilience toward immune response, and a stabilized chemical environment (5, 7, 15, 20–23). In an infectious regime, detached, colonizing single cells may be more vulnerable than aggregates (24, 25). Thus, aggregates may provide bacteria with a protected mode of colonization of new niches in a hostile environment (15, 22). In fact, in *ex vivo* samples from chronic infections, single cells are rarely observed; instead, nonattached aggregates seem to be the norm (20, 26–28).

### Conclusion.

In conclusion, our results show that aggregates perform better than single cells during biofilm development when the biofilm is seeded at a high cell density, corresponding to high initial competition, and that over long time scales, biofilm structures are likely to become dominated by progeny originating from aggregates. Our results call for a revision of the prevailing picture of *in vitro* biofilm development to consider the role played by biofilm seeding by preformed aggregates. While our study has focused on the role of spatial structure in the development of bacterial biofilms, 3D growth of multicellular assemblies is a universal phenomenon within biology, from carcinogenesis to plant development. Therefore, the phenomenon identified here, involving the interplay between the spatial structure of the growing cell assembly and of the surrounding growth resource field, may have wider implications for other biological processes and for understanding multicellular assembly in general.

## MATERIALS AND METHODS

### Bacterial strains.

All *P. aeruginosa* strains used in this study were in a PAO1 background, which was obtained from the University of Washington, Seattle, USA. To enable visualization with confocal microscopy, we tagged wild-type (WT) *P. aeruginosa* PAO1 with green fluorescent protein (GFP) by Tn*7* transformation as described previously (29).

### Growth conditions.

We streaked all strains from frozen stock onto lysogeny broth (LB) (Fisher Scientific, USA) agar plates and incubated them overnight at 37°C. Colonies were inoculated into LB broth (Fisher Scientific, USA) and grown, shaking, overnight at 37°C. We determined the optical density at 600 nm (OD_600_) of the overnight culture using a spectrophotometer (Genesys, USA), and the culture was then adjusted to the desired OD by dilution into M9 minimal medium (Serva, Germany) with 10% (vol/vol) A10 phosphate buffer [0.15 M (NH_4_)_2_SO_4_, 0.42 M Na_2_HPO_4_ * H_2_O, 0.22 M KH_2_PO_4_, and 0.51 M NaCl; pH 6.7]. We supplemented the growth medium with 0.3 mM glucose (Fisher Scientific, USA) as a carbon source. The resulting bacterial suspensions, which contained a mixture of aggregates and single cells, were then used to initiate the growth of biofilms. To test whether glucose is the limiting resource, we also carried out experiments using 0.003 mM and 30 mM glucose.

We grew biofilms in standard flow cells by the method of Tolker-Nielsen and Sternberg (30) with the modifications of Hutchison et al. (29). We filled the flow cell system with preheated (37°C) growth medium as described above, and each of the three independent chambers of the flow cell was inoculated with 150 to 250 µl of diluted bacterial culture. We inoculated the flow cell by injecting bacterial culture into each chamber using a Luer-Lock connector. We left the inoculated flow cell alone for 1 h to allow bacteria to attach to the glass coverslip before flow was started. We maintained a laminar flow at 3 ml h^−1^ with a Watson-Marlow 205 S/CA pump (Watson-Marlow, USA) for the duration of the experiment. Once flow was started, any remaining suspended cells or aggregates were removed from the system and did not contribute significantly to biomass accumulation.

### Imaging biofilm growth.

Biofilms were grown and observed *in situ* on a confocal microscope (Zeiss Imager.Z2 microscope with LSM 710 CLSM running Zeiss Zen 2010v. 6.0 [Zeiss, Germany]) for qualitative analysis and an inverted confocal microscope (Olympus FV1000 microscope, running Fluoview 3.1a software [Olympus, Japan]) for quantitative measurements of a time series of z-stacks. For the latter, a programmable motorized stage was employed to cycle between several locations (*n* = 10 to 35) in each of three independent sample chambers. This allowed us to study the growth dynamics of regions of the flow cell with different initial conditions in the same experiment, with adequate statistics for each initial condition, and allowed us to identify confidently which location initially contained an aggregate and which contained single cells only. The microscope stage area was enclosed in an incubator chamber that maintained a constant temperature of 37°C.

Z-stacks of regions that initially contained a preformed aggregate and/or one or more single cells were recorded every 3 h, using 488-nm excitation and a 505- to 525-nm emission filter. The growth of biofilms in flow cells is typically measured by measuring biomass (30, 31–33). We used the free, open-source software ImageJ (National Institutes of Health, USA) to crop images to separate aggregates and their descendants from single cells and their descendants. Biomass was then measured and described in terms of pixels cubed, or voxels, in MatLab (MathWorks, USA) using in-house code (29). Growth in biomass as a function of time could be fitted with an exponential function. The specific growth rate, μ, is given by the exponent in the expression biomass = *Ae*^μ*t*^, where *A* is a biomass at time zero (*t*_0_) and *t* is time. μ represents the growth rate per unit of biomass and can be used as a proxy measure of cell fitness (which is ultimately a cell’s ability to propagate its genes to future generations [34]).

In this work, we classified seeding structures as aggregates only if they were at least 5 µm high and had a volume at least 10 times that of a single cell. Structures intermediate in size between this and single cells were excluded from our analysis. Because we monitored biofilm growth continuously under the microscope, we would have been able to identify attachment of new cells to the growing biofilm from the overlying growth medium. This was not observed, either for aggregates or single cells.

### Effect of elevated position on growth.

We examined the effect of height above the surface on the growth rate of cells within the biofilm. We broke glass coverslips and selected shards that had a sharp, pointed tip to reduce the influence of fluid flow around the shard and that were small enough to fit inside the chamber. Using silicone sealant (3M, USA), we placed a shard in the sample chamber such that the tip pointed opposite to the direction of flow. Bacteria that attached to the shard were positioned about 100 µm above the surface of the sample chamber and were expected to experience similar hydrodynamic conditions as those experienced by cells positioned at the very top of an aggregate. The net discharge flow in the sample chamber is laminar, with a Reynolds number of ~3 describing effective transport integrated over the entire chamber. Because laminar flow results in a fluid speed that varies with distance above the surface, we also calculate Reynolds numbers at specific locations important to our experiments: the Reynolds numbers for cells on top of the shard, on top of a typical aggregate, and 1 µm off the surface are about 50, 30, and 0.03, respectively. These Reynolds numbers are consistent with nonturbulent flow. For “shard” experiments, the inoculation and flow conditions were unchanged from our other experiments. The imaging was modified slightly in that a 40× objective (Olympus, USA) was used rather than a 60× or 100× objective. The lower-magnification objective had a greater working distance, which facilitated imaging 100 µm into the sample chamber. The analysis of images to determine growth rate was performed as described above.

### Horizontal oxygen gradients.

We measured oxygen concentration in media as it entered and left the inoculated flow cell with two flowthrough sensor cells and the FireStingO2 (Pyroscience, Germany). Measurements were taken every 60 s. This provided us with time-resolved information on the percent oxygen saturation of the ingoing and outgoing media, and thereby also allowed quantification of the drop in oxygen across the flow cell as a function of biofilm growth time.

### Computer simulation algorithm.

We used the agent-based microbial simulation package iDynoMiCs (35) to model the growth of biofilms that were seeded from preformed aggregates and single cells. In iDynoMiCs, bacterial cells are represented as particulate agents that grow and divide as a result of consumption of nutrients (see supplemental material). The growth and division processes lead to local mechanical stresses within the biofilm that are relieved by a “shoving” algorithm. In the simulations, nutrients are represented by concentration fields, which change as a result of consumption and diffusion from above. These processes give rise to local concentration gradients that can strongly influence the growth dynamics and morphology of the developing biofilm (4, 36–39). As is common in computational biofilm studies (19, 35, 39–43), our simulations were performed in two dimensions (2D) for reasons of computational efficiency. However, previous work shows that similar results are likely to be obtained in 3D simulations (14).

In our simulations, microbial growth kinetics were modeled using the Monod growth equation

$$\frac{dX}{dt}=\mu_{\max}\frac{\left[O\right]}{K_{O}+[O]}[X]$$

where [*X*] is the local concentration of biomass, [*O*] is the local concentration of oxygen, *μ*_max_ is the maximum specific growth rate, and *K_O_* is the concentration of oxygen at which the growth rate is half the maximum (see supplemental material). The growth parameters *μ*_max_ and *K_O_* were assumed to be the same for all cells (i.e., both aggregated and single cells). We used growth parameters from previous empirical studies on *P. aeruginosa*, with oxygen as the single rate-limiting nutrient (Table 1). A bulk oxygen concentration of 6.64 × 10^−3^ g liter^−1^ (44) was used in all simulations, consistent with the saturation concentration of oxygen in water at 37°C. Using these parameters, our simulations produce biofilms several hundred micrometers in height after 120 h of growth (see Fig. S1 in the supplemental material).

**TABLE 1:** Parameters used in all agent-based simulations of biofilm growth

Parameter	Description	Value	Note and/or reference(s)
[*N*]_bulk_	Bulk concn of limiting growth resource	6.64 × 10^−3^ g liter^−1^	Saturation concn of oxygen in water at 37^o^C (44)
*Y_X/O_*	Yield coefficient of biomass per gram of oxygen consumed	0.64 g g^−1^	45
*μ*_max_	Maximum specific growth rate	0.29 h^−1^	45
*K_O_*	Half saturation concn of oxygen	8.12 × 10^−4^ g liter^−1^	Beyenal et al. (45) use Tessier kinetics to describe the growth of *P. aeruginosa* on glucose and oxygen. We assume oxygen to be rate limiting and therefore relate the constant *k_O_* (1.18 × 10^–3^ g liter^–1^) in their kinetic expression to our *K_O_* in the Monod expression via *K_O_* = *k_O_* ln2. For derivation of this expression, see the supplemental material.
ρ_B_	Density of bacteria	200 g liter^−1^	20, 40, 46
*D_O_*	Diffusivity of oxygen in water	2.3 × 10^−4^ m^2^ day^−1^	37
*L_x_*	Length of system in the vertical direction	1,032 µm	Ensures aggregates do not interact periodically
*L_y_*	Length of system in the horizontal direction	1,032 µm	Ensures aggregates do not interact periodically
*L*_DBL_	Length of diffusion boundary layer	80 µm	Within range of values from references 40, 47, and 48

### Creation of initial simulation configurations.

To create the initial configurations for our simulations, circular aggregates of cells were generated by excision of an ~100-cell circular region from a biofilm that had previously been simulated (see supplemental material). This aggregate was placed on a surface and surrounded by single cells, at a given surface density, and placed at random on regions of the surface that were not occupied by the aggregate (see Fig. S1A in the supplemental material). In the simulations presented here, the single cells on the surface form a layer that is only approximately one cell thick even at our highest density.

To ensure adequate statistical sampling of aggregate configurations, four independent aggregate configurations were generated, each of which was simulated for 10 independent realizations of the distribution of surrounding single cells on the surface for each cell density. Thus, each simulation data point represents results averaged over 40 different simulations.

### Simulation runs.

For each value of the surface density of the single cells, we simulated up to 120 h of biofilm growth starting from each of our 40 cell configurations. The growth of the aggregate, in terms of number of progeny per initial aggregated cell, was computed for each simulation run, and the results were averaged over the 40 runs.

To assess the fitness of the single cells, initially seeded on the surface, we performed separate simulations in the absence of the aggregate, for each value of the single-cell density. Analyzing single-cell and aggregate fitness in separate simulations mimics our experimental scenario, in which the growth of the initially unaggregated cells was monitored in regions many fields of view away from an aggregate. Cell growth, in terms of number of progeny per initial single cell, was also measured in these simulations.

For a control, we also performed simulations in which the height advantage of the aggregate was eliminated. To do this, we surrounded the aggregate with a high density of surrounding cells on the surface (0.5 cell µm^−1^) and disabled the growth of the aggregate until the surrounding cells grew to the height of the aggregate (see Fig. S7A in the supplemental material). At this point, we enabled growth of the aggregate cells and ran the simulation for 120 h. To assess the success of the aggregate versus the single cells, we subtracted the time it took for the single unaggregated cells to reach the same height of the aggregate (15 h) from 120 h. The “fitness” measure for the red cells, again assessed in separate simulations, was then given by *N*_105_/*N*_0_ where *N*_105_ is the biomass after 105 h.

### Statistical analysis.

Statistical significance of both experimental and simulation data was evaluated by a Mann-Whitney test. *P* values of <0.05 were considered significant. All tests were performed in GraphPad Prism 5 (GraphPad Software, USA).

## SUPPLEMENTAL MATERIAL

Text S1  Supplemental materials and methods with additional information about *in vitro* flow cell experiments using different glucose concentrations in the media to determine whether glucose was a limiting growth factor, as well as additional information regarding the generation of circular aggregates for *in silico* simulations and the *in silico* simulations in general. Download Text S1, PDF file, 0.5 MB

Figure S1  Simulation snapshots of biofilms seeded with a bacterial aggregate generated from a pregrown biofilm. (A) Biofilm development involving initially aggregated cells (green) and surrounding single cells (red) at a low density (0.01 cell µm^−1^). The aggregate is also magnified (blue region) for purpose of visualization. (B) Generating bacterial aggregates of circular geometry. For the purpose of visualization, the radius *R* in the schematic is much larger than the 20 µm that was actually used. Download Figure S1, TIF file, 0.6 MB

Figure S2  Whether aggregates or single cells produce more progeny in the first 120 h of growth depends on the starting density of cells. Shown are linear regressions based on simulated growth of either single cells or cells in an aggregate. *N*/*N*_0_ gives the number of progeny per original cell as a function of growth time. (A) For a low-density inoculum (0.01 cell µm^−1^), the slope of *N*/*N*_0_ for aggregates is 2.247 ± 0.02149 h^−1^ and the slope of *N*/*N*_0_ for single cells is 37.77 ± 0.2769 h^−1^. Here, aggregates grow faster than single cells. The growth of both aggregates and single cells fit well as linear functions of time (*r*^2^ = 0.9934 and *r*^2^ = 0.9993). (B) For a high-density inoculum (0.5 cell µm^−1^), the slope of *N*/*N*_0_ for aggregates is 1.377 ± 0.01345 h^−1^ and the slope of *N*/*N*_0_ for single cells is 0.7409 ± 0.0004667 h^−1^. Thus, here, aggregates grow faster than single cells. Note that the vertical axis in panel B covers a much smaller scale than in panel A. As described above for panel A, the growth of both aggregates and single cells fit well as linear functions of time (*r*^2^ = 0.9931 and *r*^2^ = 1.000). Download Figure S2, TIF file, 0.03 MB

Figure S3  Two examples of the large structures resulting from a preformed aggregate of GFP-tagged *P. aeruginosa* after 99 h of growth in a flow cell. (A and C) Cross section/top-down view of two aggregates. (B and D) 3D projections of two aggregates. Magnification, ×630. Download Figure S3, TIF file, 0.2 MB

Figure S4  The relative fitness of the aggregate increases with increasing competition. We measure the relative fitness of the aggregate as the ratio of the number of progeny per initial cell (*N*/*N*_0_) for an aggregate to the number of progeny per initial cell for single cells. Thus, ratios greater than 1 indicate that the aggregate is fitter than the single cells. The relative fitness of the aggregate at 10, 30, and 120 h is plotted as a function of the initial density of cells on surrounding cells. Download Figure S4, TIF file, 0.01 MB

Figure S5  Schematic drawing of the flow chamber with a 100-µm glass platform with single cells attaching on the surface (green) and on top of the platform (blue). Download Figure S5, TIF file, 0.2 MB

Figure S6  Exponential growth rate during the first 9 h of growth for aggregates or single-cell population of *P*. *aeruginosa* PAO1 in M9 minimal medium supplemented with either 0.3 or 30 mM glucose at an initial cell density of OD of 0.01. Values are means ± SEM (error bars). Download Figure S6, TIF file, 0.02 MB

Figure S7  The aggregate produces fewer progeny per initial cell relative to that of the single cells when its height advantage is eliminated. (A) Growth represented by *N*/*N*_0_ for aggregates and single cells grown at the same height in computer simulations. Single-cell growth was measured at the same height as aggregates over 105 h. The initially unaggregated single-cell population produces significantly more progeny per initial cell than the aggregate. Values are means ± SEM (error bars). (B) Simulation snapshot at 15 h of single-cell growth (aggregated growth switched off). At 15 h, the initially unaggregated competing cells (red) have reached the same height as the aggregated cell population (green). Download Figure S7, TIF file, 0.1 MB

Table S1  Exponential growth rates and *N*/*N*_0_ of aggregates and single cells at initial densities of OD of 0.001, 0.01, or 0.1. The values are shown as means and as standard deviations (SD). The *P* values shown compare the values for aggregates and single cells by the Mann-Whitney test. Table S1, DOCX file, 0.02 MB

Table S2  Exponential growth rates of single cells on the surface or on a step 100 µm above the surface at initial cell densities of OD of 0.1, 0.01, or 0.001. Mean exponential growth rates and standard deviations (SD) are shown. The *P* values shown compare the values for surface and step populations by the Mann-Whitney test. Table S2, DOCX file, 0.01 MB
