# Supplementary material for: Role of Multicellular Aggregates in Biofilm Formation
Source: mBio. 2016 Mar 22;7(2):e00237-16. doi: 10.1128/mBio.00237-16 (PMC4807362; doi:10.1128/mBio.00237-16)
Supplement: Text S1 — Supplemental materials and methods with additional information about in vitro flow cell experiments using different glucose concentrations in the media to determine whether glucose was a limiting growth factor, as well as additional information regarding the generation of circular aggregates for in silico simulations and the in silico simulations in general. Download [file mbo002162738s1.pdf]

Supplementary materials

The limiting growth substrate may be oxygen

When biofilms are grown in flow cells, steep chemical gradients in the concentration of carbon sources, trace metals, and oxygen can arise (4, 29–32). Our media contains glucose as the sole carbon source and oxygen as the electron acceptor. Thus, as bacteria require a carbon source and electron acceptor for growth, competition for one or both of these resources should drive the enhancement of the aggregates' relative fitness. To determine what the limiting growth resource is, we varied the glucose concentration over four orders of magnitude (0.003 mM to 30 mM), at our medium-competition density of cells, corresponding to an inoculum of OD = 0.01. If glucose were the limiting resource we would expect that changing glucose concentration by a factor of 10,000 should change the relative fitness of aggregates with respect to single cells. Instead, we found that there was no difference in growth rates between aggregates and single cells regardless of the glucose concentration ($P = 0.4525$; Figure S7). These results indicate that glucose is not a limiting resource in our experiments.

Generation of Circular Aggregates

To generate bacterial aggregates to seed the surface in the simulations, circular areas containing approximately 100 cells were cut out from previously grown (simulated) biofilms (see Figure S1B). The centre of the circular area was chosen in the middle of the biofilm to ensure no overlaps between neighbouring bacteria, and to ensure that there was a uniform number density of bacteria in the aggregate. The aggregate was generated by computing all bacteria that lay within a radius R = 20 µm of the centre O (Figure S1B). This procedure gave rise to approximately 100 cells in the aggregate. The resulting aggregate was then placed on the surface as shown in Figure (S1A). Surrounding cells were then inserted at random positions excluding the region occupied by the aggregate. The initial number of these competitor cells either side of the aggregate were determined by the specified surface density (cell μm^{-1}).

Supplementary Material on Simulation Algorithm and Rate Equations

The role of multicellular aggregates in biofilm formation

Simulation implementation

To model biofilm growth, starting from configurations such as those shown in Figure S1, we use the agent-based microbial simulation package iDynoMiCs [1]. In iDynoMiCs, individual bacteria, represented as spheres (or discs in 2D), grow at a rate that is dependent on the local nutrient concentration, which is in turn altered by the resulting growth dynamics of the bacteria. Consumption of the nutrients by bacteria, leads to growth and proliferation of the microbial community, giving rise to local stresses that are subsequently alleviated via inter-cellular "shoving". During each global time-step of the simulation, the order in which cells are selected to grow and divide is stochastic. The location of each daughter cell within the area surrounding the mother cell upon division is also computed stochastically.

In the simulation, nutrient is assumed to diffuse towards the biofilm from a bulk region far from the top of the growing biofilm, with the concentration of nutrient in this bulk region set to a constant value. In the biofilm region, the diffusion of nutrients is hindered relative to regions outside the biofilm. Periodic boundary conditions are imposed on both the nutrient concentration field and the particle coordinates in the horizontal direction, while the condition of zero-flux is imposed at the surface.

Mathematically, the dynamics of the nutrient, which is represented as a

concentration field, are governed by the reaction-diffusion equation

$$\frac{\partial O(\mathbf{x})}{\partial t} = \nabla \cdot (D_O(\mathbf{x}) \cdot \nabla O(\mathbf{x})) + r_O(\mathbf{x}), \tag{S1}$$

where $O(\mathbf{x})$ is the space ($\mathbf{x}$)-dependent oxygen concentration, $D_O(\mathbf{x})$ is the diffusion coefficient of oxygen, and $r_O(\mathbf{x})$ is the consumption rate of the oxygen by the bacteria. The rate of oxygen consumption, $r_S(\mathbf{x})$ is related to the growth rate of the bacteria, $\mathrm{d}X/\mathrm{d}t$, via

$$r_O(\mathbf{x}) = \frac{\mathrm{d}S}{\mathrm{d}t} = -\frac{1}{Y_{X/O}} \frac{\mathrm{d}X}{\mathrm{d}t}. \tag{S2}$$

$X(\mathbf{x})$ is the local biomass density, and $Y_{X/O}$ is the yield coefficient that describes the amount of substrate required to produce one unit of biomass, X.

The growth rate of each bacterium is governed by the equation

$$\frac{\mathrm{d}X}{\mathrm{d}t} = \mu_{max} \frac{O}{K_O + O} X. \tag{S3}$$

This is the well-known Monod function for bacterial growth. Here, μ_{max} is the maximum specific growth rate of the bacteria, and K_O is the concentration of oxygen, O, at which the growth rate is half maximal. In our simulations, kinetic growth constants from empirical and simulation studies on *Pseudomonas aeruginosa* were used as input growth parameters. Note that the growth rate parameters $Y_{X/O}$, μ_{max}, and K_O are the same for both the aggregate cells and the surrounding competitor cells on the surface (see Table 1 of main manuscript).

It is practical, due to the different timescales for oxygen diffusion and cell growth, to assume a pseudo-steady state oxygen concentration with respect to biomass growth. Therefore one can remove the time dependency in Equation S1 to give

$$0 = \nabla \cdot (D_O(\mathbf{x}) \cdot \nabla O(\mathbf{x})) + r_O(\mathbf{x}). \tag{S4}$$

Obtaining K_O for Monod kinetics from k_O Tessier kinetics

Bayenal *et al* describe the growth of Pseudomonas *aeruginosa* using dual substrate Tessier kinetics [1]

$$\mu = \mu_{max}(1 - e^{-s_O/k_O})(1 - e^{-s_G/k_G}), \tag{S5}$$

where s_G and s_O are the glucose and oxygen concentrations respectively, and k_G and k_O are constants. The constant k_O in Equation S5 is not the same as the K_O (the concentration of oxygen, O, at which the growth rate is half maximal) in Equation S3. In the main manuscript we set $K_O = k_O \ln 2 = 8.12 \times 10^{-4}$ g L^{-1}. To derive the expression $K_O = k_O \ln 2$, we start by assuming that $s_G >> k_G$ in the Tessier equation above, i.e., glucose is in abundance. Therefore the factor $(1 - e^{-s_G/k_G}) \to 1$, giving

$$\mu = \mu_{max}(1 - e^{-s_O/k_O}). \tag{S6}$$

The growth rate is half maximal at an oxygen concentration of K_O

$$\frac{\mu_{max}}{2} = \mu_{max}(1 - e^{-K_O/k_O}) = \frac{1}{2}(1 - e^{-K_O/k_O}), \tag{S7}$$

which when solved for K_O gives

$$K_O = k_O \ln 2. \tag{S8}$$

We use Equation S8 to obtain the value of K_O in Equation S3.

References

[1] Lardon, L. A., Merkey, B. V., Martins, S., Dtsch, A., Picioreanu. C., Kreft, J.-U., & Smets, B.F., iDynoMiCS: Next-Generation Individual-Based Modelling of Biofilms. *Environ. Microbiol.* **13**, 2416-2434 (2011)

[1] Bayenal, H., Suet, N. C., & Lewandowski, Z., The Double Substrate Growth Kinetics of Pseudomonas *aeruginosa Enzyme Microb. Technol.* **32**, 91-98 (2003)
