# Supplementary figures and images for: Role of Multicellular Aggregates in Biofilm Formation
Source: mBio. 2016 Mar 22;7(2):e00237-16. doi: 10.1128/mBio.00237-16 (PMC4807362; doi:10.1128/mBio.00237-16)

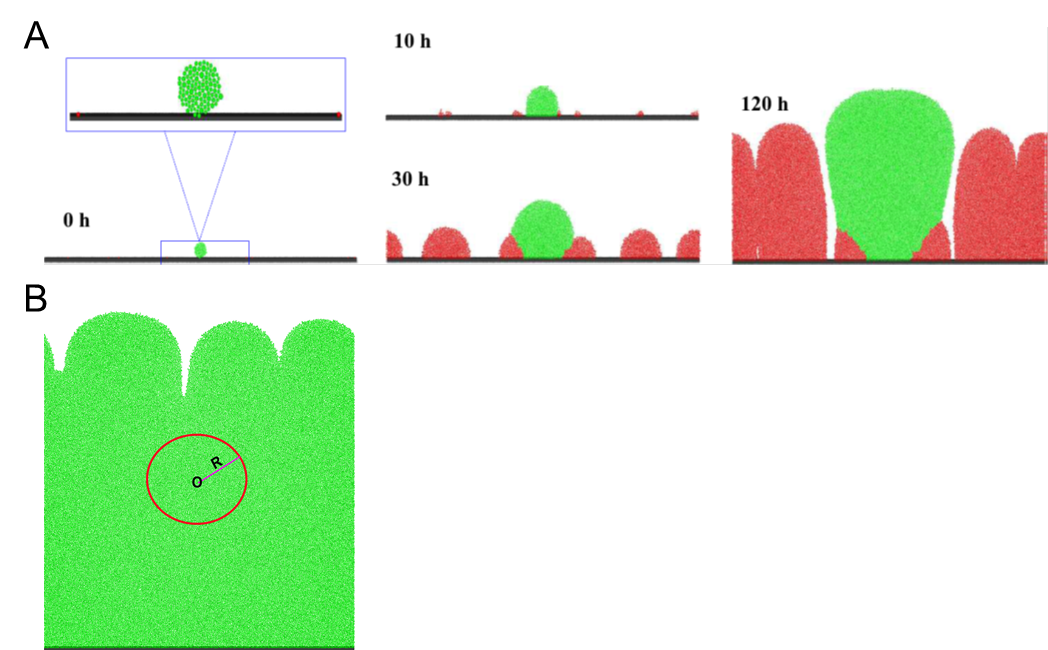

Supplement: Figure S1 — Simulation snapshots of biofilms seeded with a bacterial aggregate generated from a pregrown biofilm. (A) Biofilm development involving initially aggregated cells (green) and surrounding single cells (red) at a low density (0.01 cell µm−1). The aggregate is also magnified (blue region) for purpose of visualization. (B) Generating bacterial aggregates of circular geometry. For the purpose of visualization, the radius R in the schematic is much larger than the 20 µm that was actually used. Download [file mbo002162738sf1.tif]

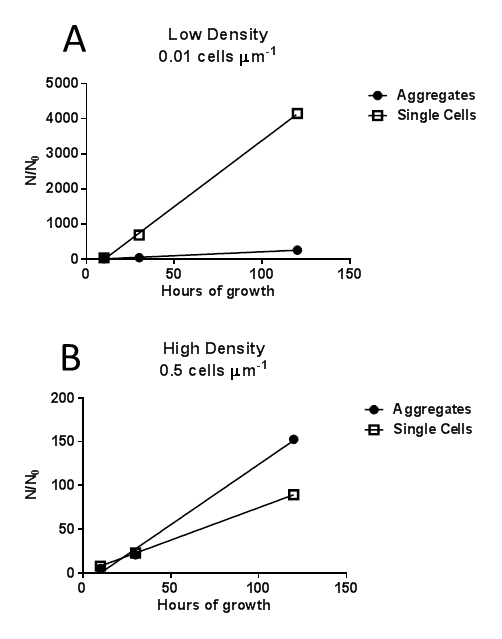

Supplement: Figure S2 — Whether aggregates or single cells produce more progeny in the first 120 h of growth depends on the starting density of cells. Shown are linear regressions based on simulated growth of either single cells or cells in an aggregate. N/N0 gives the number of progeny per original cell as a function of growth time. (A) For a low-density inoculum (0.01 cell µm−1), the slope of N/N0 for aggregates is 2.247 ± 0.02149 h−1 and the slope of N/N0 for single cells is 37.77 ± 0.2769 h−1. Here, aggregates grow faster than single cells. The growth of both aggregates and single cells fit well as linear functions of time (r2 = 0.9934 and r2 = 0.9993). (B) For a high-density inoculum (0.5 cell µm−1), the slope of N/N0 for aggregates is 1.377 ± 0.01345 h−1 and the slope of N/N0 for single cells is 0.7409 ± 0.0004667 h−1. Thus, here, aggregates grow faster than single cells. Note that the vertical axis in panel B covers a much smaller scale than in panel A. As described above for panel A, the growth of both aggregates and single cells fit well as linear functions of time (r2 = 0.9931 and r2 = 1.000). Download [file mbo002162738sf2.tif]

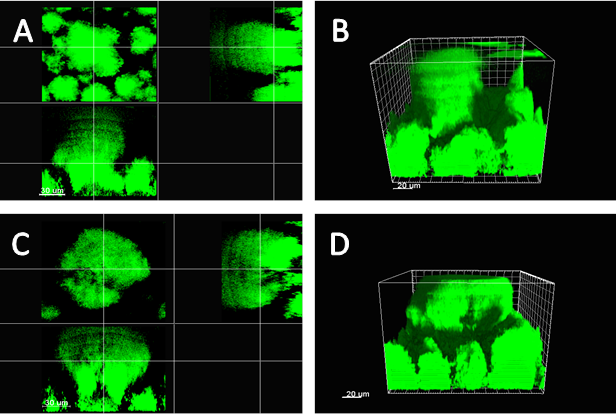

Supplement: Figure S3 — Two examples of the large structures resulting from a preformed aggregate of GFP-tagged P. aeruginosa after 99 h of growth in a flow cell. (A and C) Cross section/top-down view of two aggregates. (B and D) 3D projections of two aggregates. Magnification, ×630. Download [file mbo002162738sf3.tif]

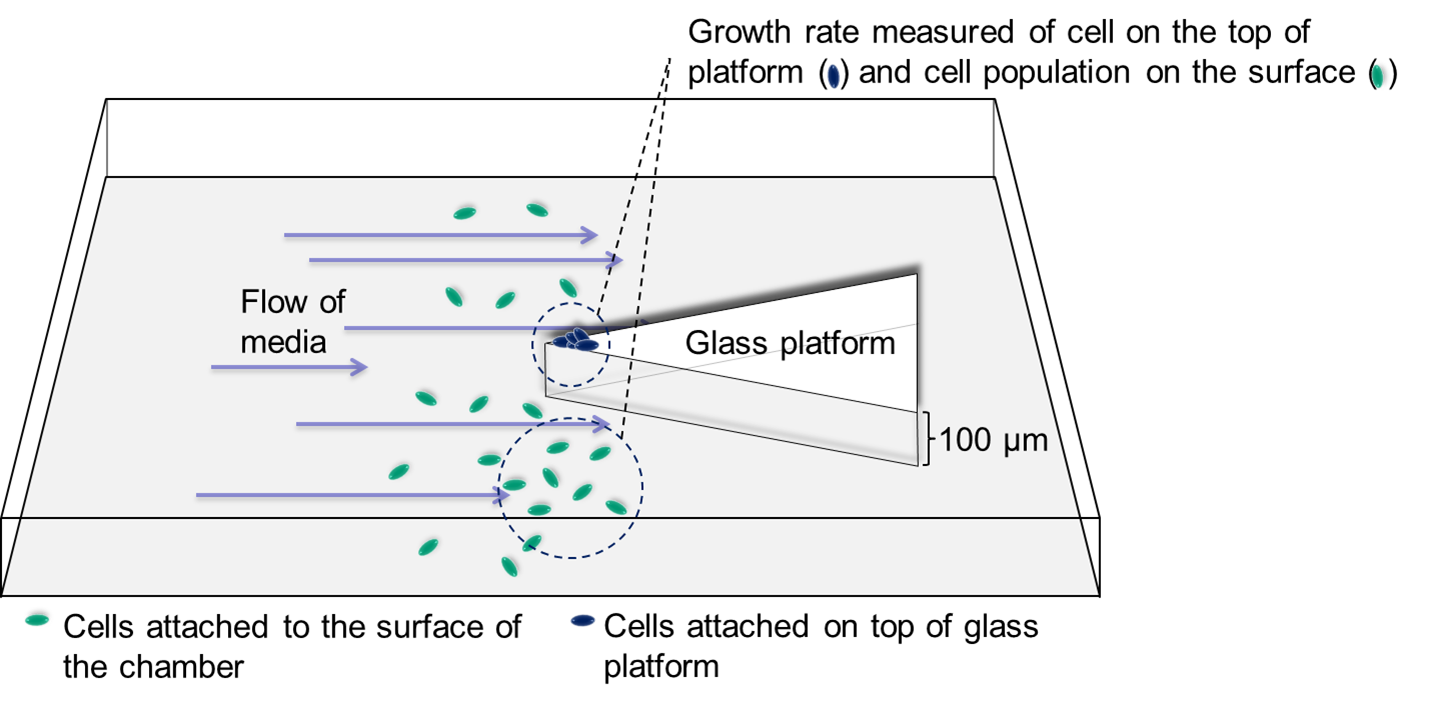

Supplement: Figure S5 — Schematic drawing of the flow chamber with a 100-µm glass platform with single cells attaching on the surface (green) and on top of the platform (blue). Download [file mbo002162738sf5.tif]

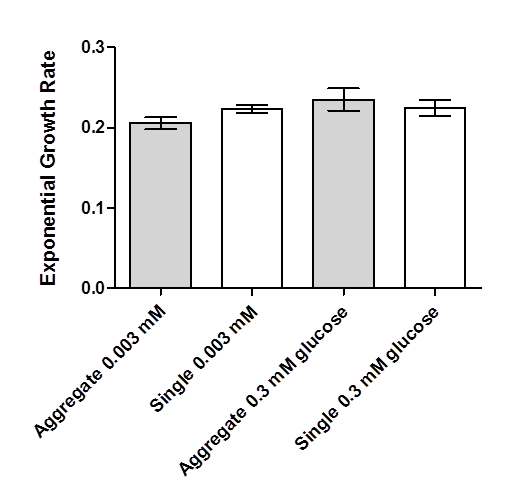

Supplement: Figure S6 — Exponential growth rate during the first 9 h of growth for aggregates or single-cell population of P. aeruginosa PAO1 in M9 minimal medium supplemented with either 0.3 or 30 mM glucose at an initial cell density of OD of 0.01. Values are means ± SEM (error bars). Download [file mbo002162738sf6.tif]

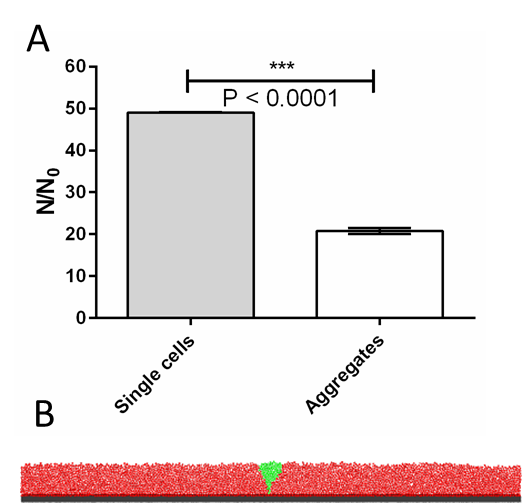

Supplement: Figure S7 — The aggregate produces fewer progeny per initial cell relative to that of the single cells when its height advantage is eliminated. (A) Growth represented by N/N0 for aggregates and single cells grown at the same height in computer simulations. Single-cell growth was measured at the same height as aggregates over 105 h. The initially unaggregated single-cell population produces significantly more progeny per initial cell than the aggregate. Values are means ± SEM (error bars). (B) Simulation snapshot at 15 h of single-cell growth (aggregated growth switched off). At 15 h, the initially unaggregated competing cells (red) have reached the same height as the aggregated cell population (green). Download [file mbo002162738sf7.tif]
