# Supplementary material for: Role of Multicellular Aggregates in Biofilm Formation
Source: mBio. 2016 Mar 22;7(2):e00237-16. doi: 10.1128/mBio.00237-16 (PMC4807362; doi:10.1128/mBio.00237-16)
Supplement: Table S1 — Exponential growth rates and N/N0 of aggregates and single cells at initial densities of OD of 0.001, 0.01, or 0.1. The values are shown as means and as standard deviations (SD). The P values shown compare the values for aggregates and single cells by the Mann-Whitney test. [file mbo002162738st1.docx]

Table S1

| **Position in flow cell** | **Initial OD** | **Mean exp. growth** | **SD** | **P-value** | **Mean N/N_0_** | **SD** | **P-value** |
| --- | --- | --- | --- | --- | --- | --- | --- |
| **Aggregates** | 0.001 | 0.1919 | 0.0126 | P < 0.0001 | 3.148 | 0.2475 | P < 0.0001 |
| **Single Cells** |  | 0.2332 | 0.0163 |  | 4.0947 | 0.6085 |  |
| **Aggregates** | 0.01 | 0.2349 | 0.0281 | P = 0.4140 | 3.98 | 0.5837 | P = 0.7679 |
| **Single Cells** |  | 0.2244 | 0.0308 |  | 4.0765 | 0.4208 |  |
| **Aggregates** | 0.1 | 0.2402 | 0.0169 | P = 0.0029 | 4.062 | 0.8569 | P = 0.0201 |
| **Single Cells** |  | 0.1795 | 0.0403 |  | 3.0108 | 0.7134 |  |
