# Supplementary material for: Role of Multicellular Aggregates in Biofilm Formation
Source: mBio. 2016 Mar 22;7(2):e00237-16. doi: 10.1128/mBio.00237-16 (PMC4807362; doi:10.1128/mBio.00237-16)
Supplement: Table S2 — Exponential growth rates of single cells on the surface or on a step 100 µm above the surface at initial cell densities of OD of 0.1, 0.01, or 0.001. Mean exponential growth rates and standard deviations (SD) are shown. The P values shown compare the values for surface and step populations by the Mann-Whitney test. [file mbo002162738st2.docx]

Table S2

| **Position in flow cell** | **Initial OD** | **Mean growth** | **SD** | **P-value** |
| --- | --- | --- | --- | --- |
| **On surface** | 0.1 | 0.1722 | 0.025 | P = 0.004 |
| **On step** |  | 0.2739 | 0.02801 |  |
| **On surface** | 0.01 | 0.2345 | 0.0065 | P = 0.0079 |
| **On step** |  | 0.3688 | 0.02089 |  |
| **On surface** | 0.001 | 0.2474 | 0.014 | P = 0.004 |
| **On step** |  | 0.3866 | 0.04483 |  |
